# Colorectal Hamartoma Presenting As a Perineal Mass in a Boy with Proximal Hypospadias

**DOI:** 10.1055/s-0040-1715182

**Published:** 2020-09-18

**Authors:** Katja P. Wolffenbuttel, Cornelius E. J. Sloots

**Affiliations:** 1 Department of Urology and Pediatric Urology, Erasmus MC Sophia, Rotterdam, The Netherlands; 2 Department of Pediatric Surgery, Erasmus MC Sophia, Rotterdam, The Netherlands

**Keywords:** colorectal hamartoma, perineal tumor, posterior hypospadias, rectal duplication

## Abstract

Congenital perineal lesions are rare and can occur along with other birth defects such as anorectal malformations (ARMs) and urogenital anomalies. A colorectal hamartoma associated with a urogenital anomaly without ARM is extremely rare. We recently treated a newborn with posterior hypospadias and a solid perineal mass diagnosed as a colorectal hamartoma.

## Introduction

Congenital perineal lesions are rare and can occur along with other birth defects such as anorectal malformations (ARMs). 1 We recently treated a newborn with hypospadias, however, without an ARM, who also had a perineal tumor based on ectopic colorectal tissue, i.e., hamartoma.

## Presentation

**Fig. 1:**
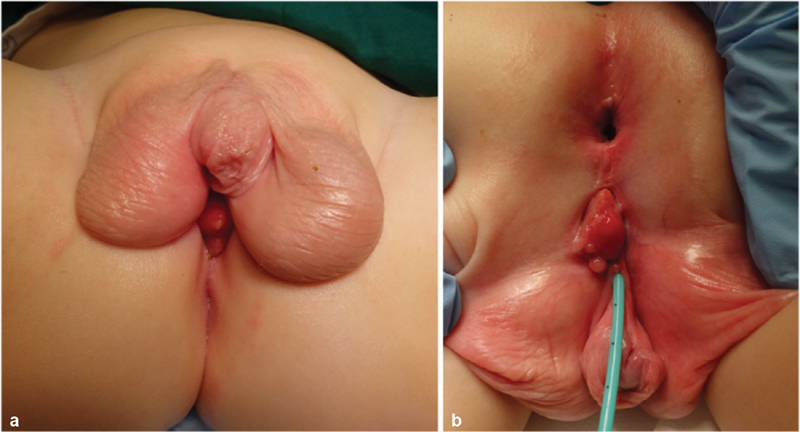
( **a** ) Photograph of the patient in supine position. ( **b** ) Preoperative photograph of the patient in prone position.

A neonate born premature at 32 weeks with a birth weight 1,740 g was evaluated for a soft dark pink perineal tumor arising 5 mm from a normal anus with associated normal passage of stools. He had a proximal hypospadias with a bifid scrotum and bilateral testes ( Fig. 1a ). Although the position of the urethral meatus could not be identified, there were no signs of urinary obstruction. Ultrasonography showed normal kidneys and urinary tract. Karyotype is 46 XY, levels of sex hormones were normal, and genetic panel for ARM and differences of sex development showed no mutations.

**Fig. 2:**
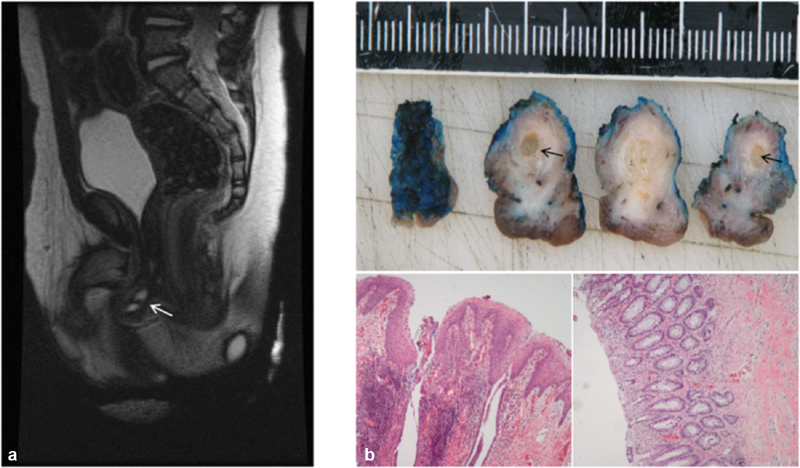
( **a** ) MRI (T2) with perineal mass with fluid-filled cavity ( *arrow* ). ( **b** ) Upper panel: macroscopic aspect with central cavity ( *arrow* ). Lower panel: hamartoma alternately lined with squamous epithelium or with colorectal mucosa. MRI, magnetic resonance imaging.

In the first 6 months the perineal lesion gradually became larger, but part from occasional slight bleeding he remained asymptomatic. At the age of 11 months he underwent a diagnostic examination under anesthesia with biopsy of the lesion. The narrow urethral meatus was found immediately adjacent to the perineal lesion ( Fig. 1b ). Cystography via an inserted suprapubic catheter showed a normal bladder, short urethra with stenotic distal part, and connection with a tubular structure posterior of the urethra. A contrast enema showed a normal rectum and colon without duplication or fistula. Magnetic resonance imaging (MRI) showed a perineal tumor of 13 × 6 × 15 mm with close approximation of the urethra, the tubular structure behind the urethra and the rectum ( Fig. 2a ). The remaining pelvic structures such as the anus, rectum, pelvic floor muscles, and anal sphincter were unremarkable. Histological examination of the biopsy showed colonic tissue.

Surgical excision was scheduled at the age of 18 months. The patient had a bowel wash-out preoperatively. Cystoscopy was performed after meatotomy of the narrow hypospadiac urethral opening and showed a normal bladder, bladder neck, and posterior urethra without evidence of an enlarged utricle, corresponding to the findings of the cystography. The tubular structure entered the bulbar urethra just below the urethral sphincter and it was blind ending. A transurethral catheter was introduced over a guidewire and a suprapubic catheter was inserted. Complete surgical excision of the perineal lesion was achieved with a dorsal approach and the patient in prone position. During the procedure, a small lesion in the perineal urethra occurred and was closed. The sphincter complex was dissected anteriorly, but the rectal mucosa remained intact. After excision of the lesion the sphincter complex was re-approximated.

The postoperative course was uneventful. Oral intake was resumed after 5 days of parenteral nutrition and the patient was discharged 6 days after surgery. Pathological examination showed a solid structure consisting of smooth muscle, partly lined with squamous epithelium and partly with colorectal mucosa and with a central cavity of 3 mm lined with cylindrical epithelium with focal goblet cells ( Fig. 2b ).

Examination under anesthesia 2 weeks postoperatively showed slightly delayed wound healing. The suprapubic tube was removed after micturition without residue. Wound healing was completed 6 weeks after surgery. After extensive consultation with the parents and the multidisciplinary team it has been decided to postpone further surgery for hypospadias correction until the patient is older.

## Discussion

Perineal lesions are uncommon and mainly occur in patients with ARM. In a series of more than 2,000 patients with ARM, the incidence of perineal lesions was 1.5%, mainly lipomas, hamartomas, and hemangiomas. 1 Although several case reports have been published, a series of perineal lesions in patients without ARM is lacking. Reports of ectopic colorectal tissue presenting as a perineal tumor in patients without ARM are even rarer. Sun et al described a neonate with hypospadias and an inverted rectal duplication presenting as a rapidly growing perineal mass connected with the pelvic diaphragm. 2 Our patient, however, is more similar to the case reported by Liu et al of a gradually enlarging perineal mass in a female infant with associated vaginal duplication. The excised perineal tumor in this case was also considered to be a hamartoma consisting of colorectal tissue, in which no connection to surrounding pelvic structures such as rectum or urethra was noted. 3

The pathogenesis of congenital colorectal hamartomas of the perineum is unknown. Liu et al mentioned that the perineum originates from the tip of the urorectal septum which separates the urogenital sinus from the anorectal tract. The close relation of these structures during embryologic development may explain the association of perineal colorectal hamartomas with developmental anomalies of the anorectal and urogenital tracts. 3 Gangopadhyay et al described two patients, the second in particular being quite similar to our patient, although the rather blurred photographs are difficult to review. They also hypothesized that an abnormal shape and development of the urorectal septum underlies this condition. 4 Perineal colorectal hamartoma should be differentiated from a bowel duplication. Duplications by definition have a close connection to a bowel segment and share a common blood supply. 5 The perineal tumor in our case was neither connected to the rectosigmoid nor with its blood supply and is therefore best classified as a hamartoma.

Diagnosis of a protruding perineal lesion must include radiologic examination using ultrasonography and MRI to delineate the extent of the lesion in relation to the surrounding structures (for example the urethra, bladder, vagina, and/or rectum) and to detect associated malformations within these structures. Contrast studies can be helpful to exclude a fistula to urogenital or colorectal structures. Biopsy to determine histology may be of additional value if diagnosis after imaging and endoscopic examination is still unclear. Treatment of a perineal lesion after establishing the precise relationship with the surrounding structures is surgical in nature. A careful dissection with special attention to nearby structures like urethra and rectum and use of bipolar coagulation is important for a successful treatment.

## Conclusion

Congenital perineal masses are mainly reported in association with ARM. Reports of perineal colorectal hamartomas without ARM are exceptional. We describe the third case in a male infant, of a perineal colorectal hamartoma associated with posterior hypospadias. Unlike rectal duplications, no connection to adjacent structures is found in perineal colorectal hamartomas. Given the close relationship of the developing perineum with the urogenital and anorectal tracts, an accurate radiological evaluation and endoscopy of the pelvic organs prior to surgical excision is recommended.

## Notes

New insights and the Importance for the Pediatric Surgeon Treating patients with rare conditions is challenging, partly because of the lack of guidance from the literature. Reporting rare case studies, such as this patient with a protruding congenital perineal mass, contributes to the quality of care for patients with a rare condition.
